# Application of avidin-biotin technology to improve cell adhesion on nanofibrous matrices

**DOI:** 10.1186/s12951-015-0096-2

**Published:** 2015-05-16

**Authors:** Jian-feng Pan, Ning-hua Liu, Lin-yuan Shu, Hui Sun

**Affiliations:** Department of Orthopaedics, Shanghai Jiao Tong University Affiliated Sixth People’s Hospital, 600 YiShan Road, Shanghai, 200233 China; Department of Emergency, Shanghai Jiao Tong University Affiliated Sixth People’s Hospital, 600 YiShan Road, Shanghai, 200233 China

**Keywords:** Electrospun nanofibers, PLCL/Pluronic, Avidin, Biotin

## Abstract

**Background:**

Electrospinning is an easy and effective technique to produce submicron fibers possessing a range of attractive characteristics such as interconnected porous structures similar to natural ECM and good resilience to movement. Rapid and efficient cell attachment to nanofibrous matrices is a necessary prerequisite in tissue engineering. Thus, the aim of this study is to evaluate poly(ε-caprolactone-co-lactide)/Pluronic (PLCL/Pluronic) nanofibrous matrices with avidin-biotin technology for improving cell adhesion for the first time.

**Results:**

PLCL/Pluronic nanofibers had relatively homogeneous fibers and interconnected porous structures. Pluronic significantly modified the hydrophilicity of nanofibrous matrices and PLCL/Pluronic nanofibrous matrices had better performance on maintaining cell proliferation. Avidin-biotin technology had no negative effect on the hydrophilic property, mechanical property and cell proliferation. Meanwhile, the attachment and spreading of adipose-derived stem cells (ADSCs) onto PLCL/Pluronic nanofibrous matrices with avidin-biotin technology was promoted obviously.

**Conclusions:**

PLCL/Pluronic nanofibrous matrices inheriting the excellent characteristics of both PLCL and Pluronic have the better cell adhesion ability through avidin-biotin technology, implying a promising application in skin care, tissue regeneration and other related area.

## Background

Skin serves as a natural barrier protecting the organism against the outside world and any break in it must be rapidly closed and efficiently repaired. Clinically, the common treatments for skin defect repair are allogeneic skin graft, mesh skin graft and split-thickness skin graft [1, 2]. However, these approaches have several shortcomings, including graft contraction, secondary donor site injury, immune rejection and graft dysfunction. Tissue engineering is considered as a promising alternative therapy to solve these problems. The basic strategy of tissue engineering is to fabricate a scaffold to mimic the natural extracellular matrix (ECM) [3].

The skin extracellular matrix consists of micron to submicron fibrils network of structural and regulatory proteins produced by encapsulated cells to form matrix architecture [4]. Electrospinning is an easy and effective technique to produce submicron fibers possessing a range of attractive characteristics such as interconnected porous structures similar to natural ECM and good resilience to movement. Additionally the thickness of electrospun membranes varies from several μm to mm which matches the depth of human skin epidermis through controlling the deposition time in the electrospinning. These advantages make the electrospun scaffolds ideal substitute for skin [5–7]. In our previous study we have fabricated poly(ε-caprolactone-co-lactide)/Pluronic (PLCL/Pluronic) blended nanofibrous matrices via electrospinning technique [8]. Among the biodegradable and biocompatible polymers commonly utilized for tissue engineering, PLCL exhibits favourable elastic and mechanical properties [9]. However, the hydrophobicity of PLCL contributes to a hydrophobic microenvironment, which is not in favor of cell adhesion. Surface hydrophilicity obtained by blending with surfactants in turn influences protein adsorption and cell attachment. Pluronic, consisting of hydrophilic poly(ethylene oxide) (PEO) and hydrophobic poly(propylene oxide) (PPO) blocks arranged in tri-block structure: PEO-PPO-PEO, is introduced to improve the hydrophilic performance of PLCL [10].

In addition to electrospun matrices, adipose-derived stem cells are promising in skin tissue engineering due to the advantages of great proliferative capacity, differentiation into epidermis and easy availability by liposuction [11]. In tissue engineering rapid and efficient cell attachment to nanofibrous matrices is a necessary prerequisite [12]. A variety of cell seeding techniques, such as gravitational seeding [13], magnetic cell seeding [14], rotational vacuum seeding [15], electrostatic cell seeding [16] and dynamic perfusion seeding [17], have been developed for the construction of tissue-engineered grafts. These techniques have several limitations that result in low seeding efficiency and minimal cell penetration into scaffolds. The natural adhesion is based on the formation of integrin-mediated bonds between adhesion proteins or motifs on matrix and integrin in the cell membrane [18]. The efficacy of cell attachment is influenced by the availability of cell membrane integrin. However, binding between the protein avidin and biotin is an extrinsic, integrin-independent receptor-ligand system which can be introduced to increase cell-seeding efficiency. This technology is based on the extraordinary affinity of avidin for biotin [19]. Avidin is a glycoprotein found in the egg white and has multiple binding sites for biotin. When used in tissue engineering, biotin is conjugated to the cell membranes and avidin is immobilized to biomaterial surfaces [20]. The highly specific binding capability between these two molecules could mediate cell attachment onto biomaterial surfaces. The bond formation between biotin and avidin is rapid and, once formed, is unaffected by extremes of pH, temperature, organic solvents and most denaturing agents. Moreover, the avidin-biotin complex is the strongest known non-covalent interaction (10^15^ M^−1^) between a protein and ligand, which is much higher than biospecific cell attachment reactions such as integrin-fibronectin (10^6^ M^−1^) or integrin-laminin (10^9^ M^−1^) [21]. Therefore Kuo has predicted that avidin-biotin binding system was superior to integrin-serum protein system in the cell adhesion strength [22].

The aim of the current study was to explore the possibility of using the avidin-biotin binding system (ABBS) and fabricating avidin-biotin-PLCL/Pluronic nanofibrous matrices for skin care application. The morphology and structure of nanofibrous matrices were investigated using scanning electron microscope (SEM), and the effect of ABBS on cell attachment and spreading was determined with adipose-derived stem cells. The present work could provide a basis for further studies or practical applications in skin care or skin regeneration.

## Results

### Fiber morphologies

**Fig. 1:**
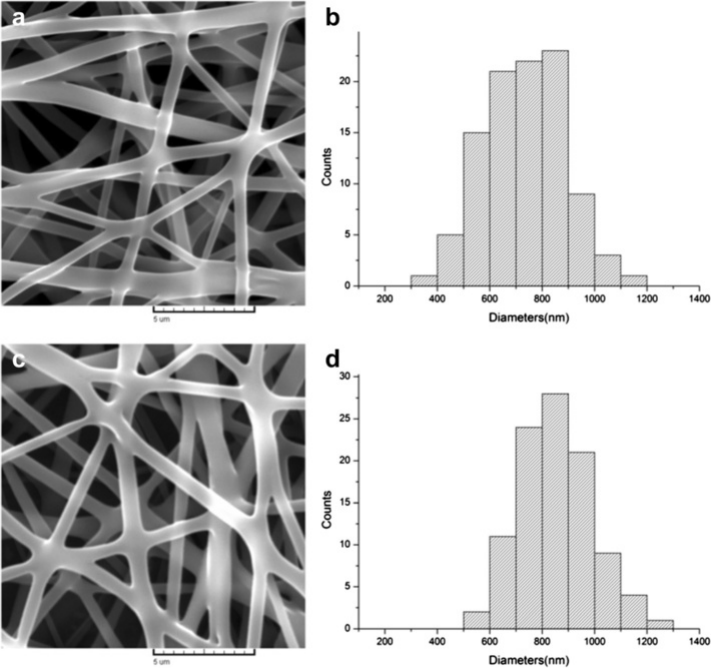
SEM images and fiber diameter distribution of electrospun PLCL (**a**, **b**) and PLCL/Pluronic (**c**, **d**) nanofibers

The SEM morphologies of PLCL and PLCL/Pluronic nanofibers were shown in Fig. 1. Smooth surface and interconnected porous structures of PLCL and PLCL/Pluronic nanofibers have been obtained. Electrospun scaffolds with microscale porous structures are most favorable for tissue engineering scaffolds because they are a network of interconnected pores that provides nutrients and gas exchange and cellular infiltration, which are crucial for cell viability and tissue regeneration. The average diameter of PLCL nanofibers from 8 wt% solution is 730.91 ± 147.64 nm, while PLCL/Pluronic nanofibers have the average diameter of 855.77 ± 137.54 nm. Fiber average diameter increased slightly with the introduction of Pluronic. At the same time, the diameter distribution histogram of nanofibers showed that the fibers had a relatively homogeneous morphology.

### Water contact angle analysis

**Fig. 2:**
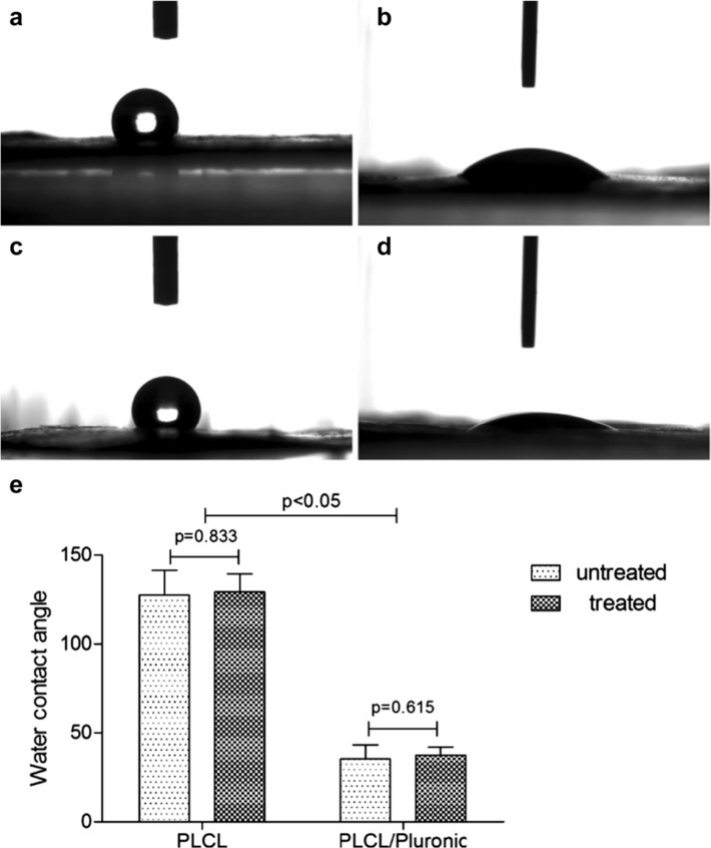
Digital pictures of water contact angles for electrospun PLCL (**a**), PLCL/Pluronic (**b**) nanofibers, avidin-treated PLCL (**c**), avidin-treated PLCL/Pluronic (**d**) and statistical analysis for differences between the electrospun scaffolds (**e**)

The surface hydrophilic property plays an important role in governing oxygen and nutrient diffusion since the tissue fluid permeates throughout the hydrophilic membranes easily. Another important parameter in hydrophilic scaffolds is that they are advantageous for efficient cell seeding and adhesion. To explore the difference of hydrophilic property between electrospun PLCL and PLCL/Pluronic nanofibers, as well as the effect of avidin on nanofibers, the water contact angle measurement was done and shown in Fig. 2. The PLCL nanofibers had a contact angle of about 127.56° ± 13.74°. It shows that PLCL is a hydrophobic polymer. In contrast, the electrospun PLCL/Pluronic nanofibers showed more hydrophilic, whose water contact angle decreased to 35.36° ± 7.85°. After avidin treated process, AB-PLCL and AB-PLCL/Pluronic nanofibers have no significant change in water contact angle (Fig. 2e, *p* > 0.05). Therefore, the introduction of Pluronic in the hybrid nanofibers resulted in the significant change of hydrophilicity and avidin has no obvious impact on hydrophilic property of nanofibers.

### Mechanical properties analysis

**Fig. 3:**
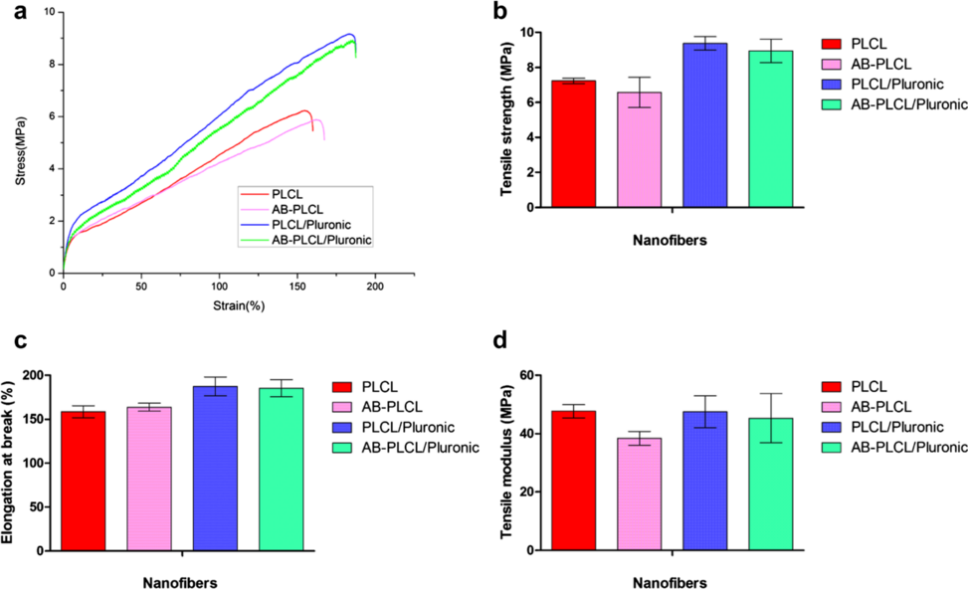
Comparison of mechanical properties for electrospun PLCL, PLCL/Pluronic nanofibers and avidin-treated groups (AB-PLCL; AB-PLCL/Pluronic). **a**: The stress–strain curves of scaffolds. **b**: Tensile strength of scaffolds. **c**: Elongation at break. **d**: Modulus

Mechanical properties of nanofibrous matrices are critical for their successful application in skin tissue engineering. Tensile tests were performed on all nanofibers to determine whether the mechanical properties were favorable for use as a skin graft. Figure 3a shows the typical tensile stress–strain curves of nanofibers under tensile loading. All scaffolds showed onset of nonlinearity in the initial stress–strain curve and the slope of the curve decreased after the onset of nonlinearity. It can be observed that PLCL/Pluronic nanofibers had the superior performance in both strength and flexibility compared with PLCL nanofibers (9.37 ± 0.38 MPa versus 7.23 ± 0.16 MPa, *p* = 0.001; 187.43 ± 10.66 % versus 158.54 ± 6.67 %, *p* = 0.003), implying that the introduction of Pluronic can improve the fiber formation and electrospinnability of blended nanofibers. Compared with natural skin grafts, PLCL/Pluronic membranes possessed comparable mechanical properties. The tensile strength and modulus of blended nanofibers is 9.37 ± 0.38 MPa and 47.49 ± 5.44 MPa, lying within the range of those of human skin (strength: 5–30 MPa; modulus: 15–150 MPa). And the elongation at break is superior to that of natural skin grafts (35 %-115 %), indicating that nanofibrous matrices are promising as a skin graft.

After avidin treated process, AB-PLCL and PLCL, AB-PLCL/Pluronic and PLCL/Pluronic nanofibers have no significant difference in tensile strength (Fig. 3b: 7.23 ± 0.16 MPa versus 6.58 ± 0.87 MPa, *p* = 0.267; 9.37 ± 0.38 MPa versus 8.95 ± 0.67 MPa, *p* = 0.393) and elongation at break (Fig. 3c: 158.54 ± 6.67 % versus 163.62 ± 4.51 %, p = 0.474; 187.43 ± 10.66 % versus 185.35 ± 9.79 %, *p* = 0.765). These results were in good accordance with water contact angle results. Thus, it is possible to prepare PLCL/Pluronic nanofibers with better mechanical property and introduce avidin-biotin binding system to promote cell adhesion on nanofibrous matrices without unfavorable mechanical effects.

### Multilineage differentiation assessment of biotinylated ADSCs

**Fig. 4:**
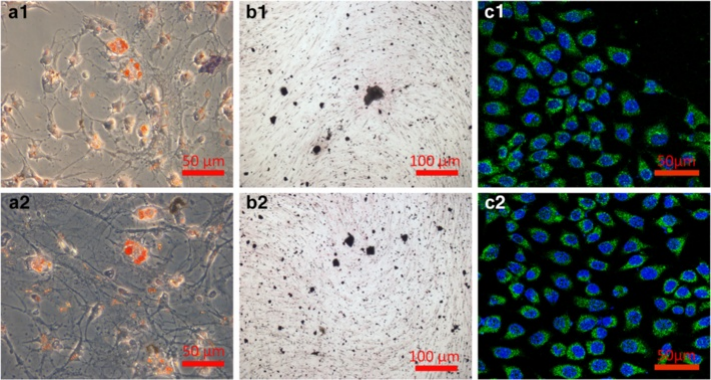
Multilineage differentiation of normal (a1, b1, c1) and biotinylated ADSCs (a2, b2, c2). **a**: After incubation in adipogenic medium cells were positively stained for adipose drops (Oil Red O); **b**: After incubation in osteogenic medium cells were positively stained for calcium deposits (Von Kossa); **c**: After incubation in epidermal induction medium cells were positively stained for cytokeratin 10 (Immunostaining)

After biotinylation process, ADSCs were incubated in lineage-specific medium to evaluate the differentiation capacity. Compared with normal ADSCs, biotinylated ADSCs still differentiated into adipocytes, osteocytes and epidermal cells (Fig. 4). Biotinylated ADSCs incubated with adipogenic medium underwent a change in their morphology from spindle shaped to intumescent and formed large adipose drops that positively stained by Oil Red O (Fig. 4a2). After incubation in osteogenic medium for 21 days, biotinylated ADSCs displayed calcium deposits stained by von Kossa, which is specific for bone mineral, a unique biochemical feature of bone mineralization (Fig. 4b2). Epidermal differentiation of biotinylated ADSCs was demonstrated by the expression of cytokeratin 10. We stained the protein a green color to assess cytokeratin 10 expression and subcellular localization. The cell nuclei were also stained by DAPI. Direct observation of the cells showed that cytokeratin 10 had a filamentous cytoplasmic distribution (Fig. 4c2). Therefore, the modification of cells with biotin does not harm the cells and affect differentiation capacity.

### Cell attachment analysis

Effective cell attachment onto scaffolds is a critical step for the fabrication of engineered tissues. Recently, avidin-biotin was found to be a powerful technology that enhanced cell attachment onto synthetic scaffolds in tissue engineering. The avidin-biotin system is widely applied due to the following advantages: (1) Highly specific affinity. It has the strongest known non-covalent interaction of a protein and ligand and allows avidin-containing molecules in a complex matrix to be bound with biotin conjugates. (2) Bridging interaction. Both of them can combine with substances having biological activity, such as protein, polysaccharides and enzymes. Therefore, the strength of the interaction and its resistance to dissociation make it easy to fabricate novel nanofibers to facilitate cell adhesion. After ADSCs incubated on the surfaces of different nanofibers for 10 min, 20 min, 30 min, 60 min and 5 h, the number of ADSCs attached to electrospun nanofibers was calculated by averaging the count of stained cell nuclei in three random fields.

**Fig. 5:**
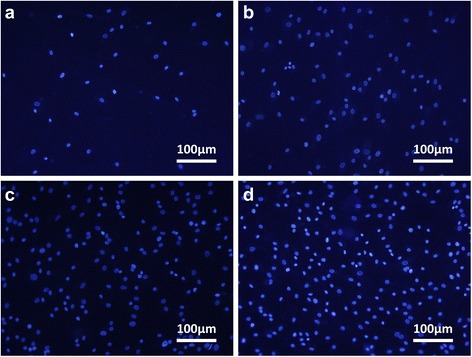
DAPI staining of ADSCs after attaching for 60 min. **a**: ADSCs + PLCL; **b**: ADSCs + PLCL/Pluronic; **c**: biotinylated ADSCs + avidinized PLCL; **d**: biotinylated ADSCs + avidinized PLCL/Pluronic

**Fig. 6:**
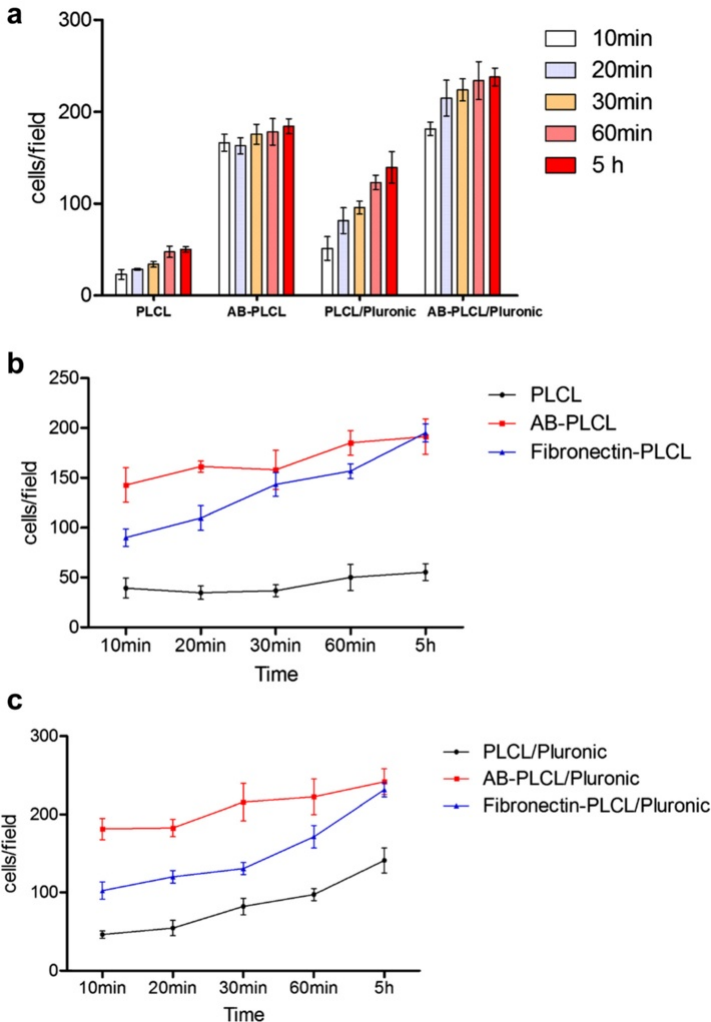
The mean number of attached ADSCs in per display window after incubation for 10 min, 20 min, 30 min, 60 min, 5 h. **a**: Cell adhesion on PLCL and PLCL/Pluronic with or without avidin-biotin binding technology; **b**: Avidin-biotin-mediated adhesion compared with integrin-mediated adhesion on PLCL; **c**: Avidin-biotin-mediated adhesion compared with integrin-mediated adhesion on PLCL/Pluronic. PLCL group: ADSCs + PLCL; PLCL/Pluronic group: ADSCs + PLCL/Pluronic; AB-PLCL group: biotinylated ADSCs + avidinized PLCL; AB-PLCL/Pluronic group: biotinylated ADSCs + avidinized PLCL/Pluronic; Fibronectin-PLCL group: ADSCs + fibronectin-treated PLCL; Fibronectin-PLCL/Pluronic group: ADSCs + fibronectin-treated PLCL/Pluronic

Figure 5 showed ADSCs stained by DAPI on the nanofibrous matrices in four groups after attaching for 60 min. The number of ADSCs attached on AB-PLCL and AB-PLCL/Pluronic was much higher than that of ADSCs on PLCL and PLCL/Pluronic by reason of avidin-biotin binding technology (Fig. 6). Compared with natural integrin-mediated adhesion, this interaction significantly increased initial cell adhesion. The results were consistent with Bhat’s study, which showed that the initial rate of cell attachment and spreading on plain glass was greater for avidin-biotin-mediated adhesion than integrin-dependent cellular adhesion [23]. Thus, avidin-biotin was introduced and bridged ADSCs and nanofibers in the process of cell adhesion.

**Fig. 7:**
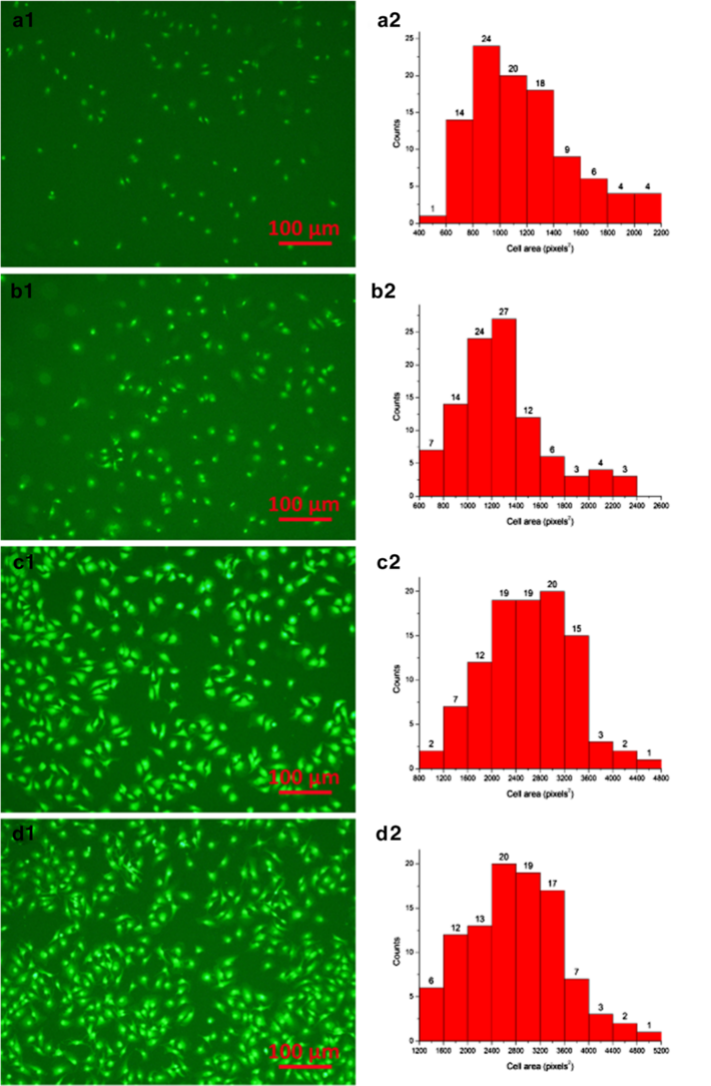
Fluorescence images and cell area distribution of ADSCs adhesion after cultivation for 5 h. **a1**, **a2**: ADSCs + PLCL; **b1**, **b2**: ADSCs + PLCL/Pluronic; **c1**, **c2**: biotinylated ADSCs + avidinized PLCL; **d1**, **d2**: biotinylated ADSCs + avidinized PLCL/Pluronic

Moreover, ADSCs on AB-PLCL and AB-PLCL/Pluronic nanofibrous matrices stretched out cytoplasmic extension to attach on fibers while PLCL and PLCL/Pluronic groups showed a round cell appearance (Fig. 7). The cell morphology was classified into three categories: round, partially spread, or fully spread. Cells with spreading area < 1200 pixels^2^ were defined as round. Cells with area between 1200 and 2400 pixels^2^ having few cytoplasmic extensions were classified as partially spread. Cells with surface area > 2400 pixels^2^ were classified as fully spread and had multiple cytoplasmic extensions in different directions. When ADSCs were seeded onto PLCL and PLCL/Pluronic, the cells failed to spread and retained a round shape. However, with the introduction of avidin and biotin, cell morphology transformed from round into spread and the percentage of round cells decreased on PLCL and PLCL/Pluronic. Therefore avidin-biotin binding system promoted cell attachment and spreading on PLCL and PLCL/Pluronic nanofibrous matrices. The focal adhesion at the contact surface is comprised of avidins as the major adhesion receptors in nanofibers and associated biotins which are the major sites of cell membrance. Furthermore, the number of ADSCs attached on PLCL/Pluronic nanofibers was higher than that on PLCL nanofibers, implying that PLCL/Pluronic nanofibers resulted in better cell adhesion than pure PLCL due to the better hydrophilic property. This is in agreement with the results of previous study by Nam [24]. Inducing the hydrophilic PEO chains to the surface of nanofibers by blended electrospinning, Pluronic significantly modified the hydrophilicity of nanofibers, which might improve cell capture efficiency and cause a change in cell attachment. With the increase of incubating time, the number of ADSCs on hydrophilic PLCL/Pluronic group increased obviously while slightly on hydrophobic PLCL group. Similarly, there was no significant difference in the count of attached ADSCs on AB-PLCL group with the increase of attaching time while attached ADSCs increased on AB-PLCL/Pluronic group. After 60 min incubation AB-PLCL/Pluronic nanofibers showed higher cell attachment rates than AB-PLCL nanofibers. Thus avidin-biotin binding technology promotes initial cell adhesion and hydrophilic property further facilitates cell adhesion with the increase of culturing time. These results exhibit that AB-PLCL/Pluronic nanofibers possess the best cell adhesion ability. We ascribe this to that the high affinity of the avidin-biotin binding system increases cell adhesion and the better hydrophilic property by introducing Pluronic is also improving cell attachment. Therefore, the high affinity avidin-biotin binding system increased cell adhesion and AB-PLCL/Pluronic nanofibers can be powerful matrices when applied for skin care application in future.

### Cell proliferation analysis

**Fig. 8:**
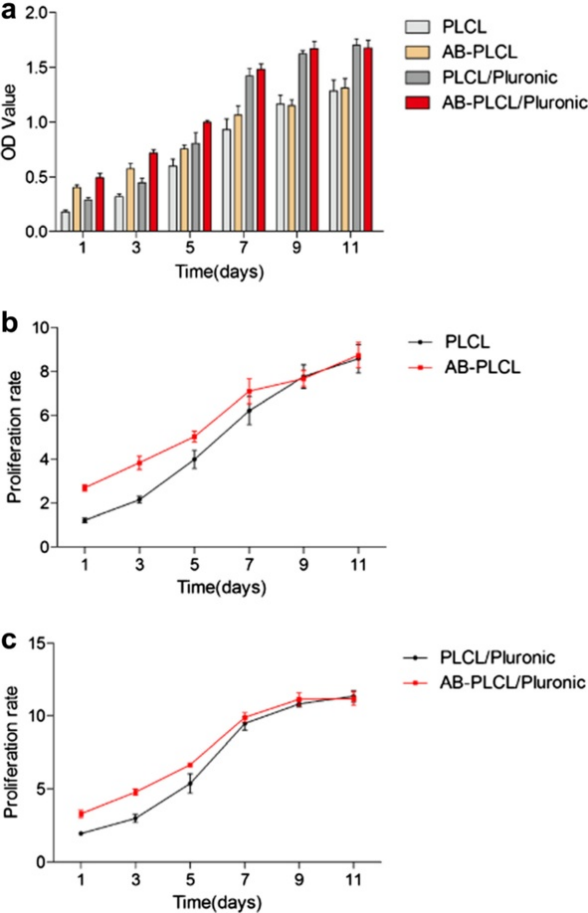
The metabolic activity and proliferation rate curve of ADSCs after cultivation for 1, 3, 5, 7, 9 and 11 days. **a**: Cell metabolic activity on PLCL and PLCL/Pluronic with or without avidin-biotin binding technology; **b**: Cell proliferation rate between PLCL and PLCL with avidin-biotin binding technology; **c**: Cell proliferation rate between PLCL/Pluronic and PLCL/Pluronic with avidin-biotin binding technology. PLCL group: ADSCs + PLCL; PLCL/Pluronic group: ADSCs + PLCL/Pluronic; AB-PLCL group: biotinylated ADSCs + avidinized PLCL; AB-PLCL/Pluronic group: biotinylated ADSCs + avidinized PLCL/Pluronic

**Fig. 9:**
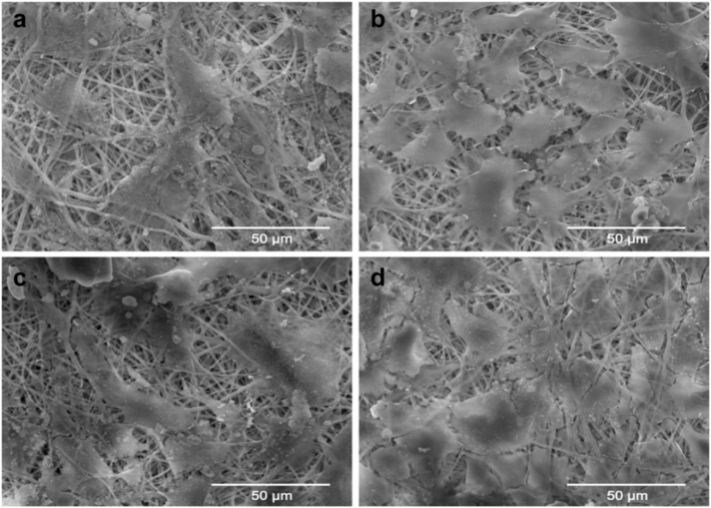
SEM images of ADSCs after cultivation for 11 days. **a**: ADSCs + PLCL; **b**: ADSCs + PLCL/Pluronic; **c**: biotinylated ADSCs + avidinized PLCL; **d**: biotinylated ADSCs + avidinized PLCL/Pluronic

Figure 8 showed the proliferation of ADSCs on the nanofibrous matrices in four groups. Figure 8a indicated that the metabolic activity increased with time in each group, implying that avidin-biotin binding system has no negative effect on cell proliferation. At day one and three, PLCL and PLCL/Pluronic with avidin-biotin binding system groups have a higher metabolic activity compared with PLCL and PLCL/Pluronic groups, which could be attributed to the improvement of cell adhesion by using avidin and biotin. Due to the initially higher number of cells cultured on these fibers, the metabolic activity in these groups is higher. Thus we normalized the metabolic activity on each day to day 0 so as to evaluate the proliferation rate between groups (Fig. 8b and c). On nanofibrous matrices treated with avidin-biotin binding system, the proliferation rate of ADSCs was higher than that of untreated groups before seven days. After that the proliferation rate of ADSCs leveled off and cell growth was equal between treated and untreated group, which is determined by the biocompatibility of nanofibrous matrices. Compared with PLCL and AB-PLCL groups, PLCL/Pluronic and AB-PLCL/Pluronic groups had higher cell growth. This was in good agreement with water contact angle results. Due to the improved hydrophilic property by introducing Pluronic, PLCL/Pluronic nanofibrous matrices have better performance on maintaining tissue fluid and governing oxygen and nutrient diffusion. Thus ADSCs cultured on PLCL/Pluronic and AB-PLCL/Pluronic group have a better proliferation rate. As showed in the Fig. 9, ADSCs on PLCL/Pluronic and AB-PLCL/Pluronic group reached a denser confluence compared with PLCL and AB-PLCL groups.

## Discussion

Tissue engineering represents an emerging field applying a set of approaches that use stem cells, bioactive agents and appropriate materials to aid tissue formation or regeneration. Several new types of biomaterials are being studied for treatment of burns, skin ulcers, deep wounds and other injuries. The PLCL copolymer, which has elastomeric properties and biodegradability, is used to fabricate electrospun matrices with nanofiber structure for skin and vascular applications. This approach allows substitute for natural skin and coverage of extremely large wounds. However, the disadvantage is the hydrophobic property against cell adhesion, which may be a prerequisite for cell growth and wound repair. Thus, blends of PLCL with Pluronic were investigated to improve the hydrophilicity of nanofibrous matrices. The water contact angle of PLCL/Pluronic blended matrices is smaller than that of PLCL matrices, indicating that the hydrophobic PLCL matrices could be transformed to a more hydrophilic state by the introduction of the Pluronic component. The surface hydrophilicity will promote the attachment, proliferation, migration and viability of many different cells. On the other hand, the mechanical properties show that PLCL/Pluronic nanofibers have the superior performance compared with PLCL nanofibers. It indicates that the introduction of hydrophilic Pluronic can improve the electrospinnability and benefit the formation of solid nanofibers. Ultimately, the tensile strength and elastic modulus of PLCL/Pluronic nanofibers is within the range of those of human skin. And the elongation at break is higher compared with that of human skin, which strengthens its potential as a skin graft, since PLCL/Pluronic could still cover the wound under a high tensile deformation.

**Fig. 10:**
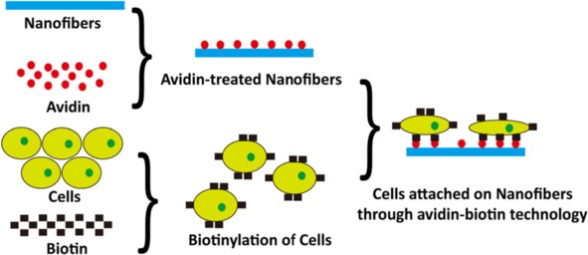
The avidin-biotin technology: the avidin was immobilized to nanofibers and the biotin was conjugated to cell membrane. The bond formation between avidin and biotin mediated cell adhesion to the nanofibrous matrices

The feasibility of introducing avidin-biotin binding system for increasing cell attachment has been heavily investigated at various levels, from molecular interactions to macroscopic responses. Tsai reported that more than 70 % of biotinylated chondrocyte adhesion to the avidin-coated surface happened within the first hour, while only approximately 32 % of normal chondrocyte adhesion occurred during the first hour [20]. The study of Kojima revealed that ABBS significantly improved initial cell attachment onto biodegradable polymer surfaces within 10 min, whereas few cells attached onto collagen-treated polymer surfaces and more time was required to form a stable adhesion complex between integrin and collagen [25]. Bhat demonstrated that the high affinity of the avidin-biotin binding system promoted initial cell adhesion and strength of cell attachment [26]. In the present study, ADSCs were modified with biotin and nanofibrous matrices were treated with avidin. The biotinylated ADSCs attached onto avidin-treated nanofibrous matrices more rapidly than the normal ADSCs attaching onto untreated matrices, and the difference of cell number between the two groups was significant. This result was consistent with the finding of Kojima mentioned above, which indicated that momentary contact might be sufficient to trap cells on surfaces when ABBS was applied. Moreover, in our case, avidin-biotin binding system was also proved to promote cell spreading on PLCL and PLCL/Pluronic nanofibrous matrices. As shown in Fig. 10, the surface of nanofibers is treated with avidin and the biotin is conjugated to cell membrane. It is easy for biotin to find avidin-containing sites to bind to when the arrest adhesion occurs. As soon as the conjugated biotin binds to the avidin molecules, a change in cytoplasmic domain of the focal adhesion occurs, which results in the transformation from ball-shaped cell appearance to flattened cell morphology.

PLCL/Pluronic nanofibers with avidin-biotin technology possess the best cell adhesion ability, which might be due to the following factors: (1) Highly specific affinity of avidin-biotin binding system. The strong non-covalent interaction of avidin and biotin facilitates biotinylated ADSCs attaching onto avidin-containing molecules in PLCL/Pluronic nanofibrous matrices. (2) The surface hydrophilicity of PLCL/Pluronic nanofibrous matrices. Hydrophilicity is an important parameter because it relates to the diffusion of nutrients and cellular waste and allows the maintaining of tissue fluid and nutrients. Adequate nutrient supply and prompt exchange of nutrition is necessary for cells to attach, migrate and proliferate.

After avidin treated process, nanofibrous matrices have no significant change in hydrophilic/hydrophobic and mechanical properties. In addition, biotinylated ADSCs still display the ability to differentiate into cell types of multiple different lineages, such as adipocytes, osteocytes and epidermal cells. The results mean that the modification of cells with biotin does not harm the cells. Therefore, PLCL/Pluronic nanofibrous matrices with avidin-biotin technology are desirable and there are reasons to believe that this artificial material is a potential alternative in clinical application.

## Conclusions

In summary, through electrospinning technology, we successfully fabricated PLCL/Pluronic nanofibrous matrices which well inherited the excellent characteristics of both PLCL and Pluronic. Avidin-biotin binding system was also utilized for increasing cell attachment. Our data indicate that the incorporation of Pluronic significantly strengthens the hydrophilicity of nanofibrous matrices. PLCL/Pluronic nanofibrous matrices possessing the high affinity avidin-biotin binding system have the better cell adhesion and proliferation capacity, implying a promising application in skin care, tissue regeneration and other related area.

## Materials and methods

### Materials

PLCL (Mn = 30,000-100,000) was purchased from ShanDong DaiGang biomaterial Co., Ltd. (China). Pluronic F-127 (Mn = 5,800), Tetrahydrofuran (THF), N, N-dimethylformamide (DMF) and fibronectin were obtained from Sigma Aldrich (St. Louis, MO, USA). Fetal bovine serum (FBS), phosphate buffered saline (PBS), Dulbecco’s modified Eagle’s medium (DMEM), penicillin-streptomycin solution, trypsin-EDTA and other culture media and reagents were purchased from Gibco Life Technologies Corporation (Carlsbad, CA, USA). CCK-8 was purchased from Dojindo Corporation (Kumamoto, Japan).

### Electrospun PLCL/Pluronic membranes

The mixed solvent of THF and DMF (v/v = 1/1) was used to prepare the electrospinning solutions by dissolving PLCL or PLCL/Pluronic (w/w = 9/1) at the concentration of 8 wt%. Electrospinning was done by using a 5 ml standard syringe with a blunt-ended needle. The syringe was located on a syringe pump and dispensed at a rate of 1.0 ml/h. A voltage of 16 kV was applied and the distance between the needle and collector was 20 cm. All electrospun fibers were deposited on the rotating collector wrapped with aluminum foil to form a thin fibrous membrane. The nanofibrous membranes were dried in vacuum at room temperature to completely remove the solvent residue.

### Fiber size analysis

To evaluate the morphology and fiber diameters of electrospun fibers, materials were gold-coated and observed using scanning electron microscope (SEM, JSM-5600LV, JEOL, Japan) at an accelerating voltage of 20 kV. For each sample (*n* = 3), five random spots were captured to generate micrographs, and at least 20 different fibers were randomly selected for further measurement using ImageJ software, version 1.46r.

### Measurement of water contact angle

To determine the influence of Pluronic on the hydrophilicity of PLCL, water contact angle test was measured using a commercial drop shape analysis system (Data Physics SCA20, Germany) in accordance with the previous study [27]. The fabric materials were cut into pieces approximately 1 × 1 cm (*n* = 6) and air-dried at room temperature for 48 h, then 3 μL deionized droplets were gently deposited on each sample through a micro syringe, images were captured at 2 s after the water droplet was dripped on the surface of materials, and the contact angle was measured by the inbuilt software in the machine.

### Tensile test

To ensure the mechanical properties of fibrous mats falls in the physiological range of human skin, mats were placed in phosphate buffered saline (PBS, Gibco, Invitrogen, USA) for 30 min and subsequently conducted following standard mechanical test. The fabric materials (200 μm in thickness) were punched into rectangular strips (70 mm × 7 mm, n = 5) and characterized by a tensile test (Instron 5567, Canton, MA). The stress–strain curves of these materials were constructed from the load-deformation curves recorded at a stretching speed of 0.5 mm/s. Ultimately the tensile strength, Young’s modulus and elongation at break were obtained from plotted stress–strain curves. Tensile property values reported here represent an average of the results for tests run on at least five samples.

### Avidin-treated nanofibrous matrices

The avidination of nanofibrous matrices was performed in accordance with the previous studies [19, 20]. Nanofibers were prepared and sterilized with ethanol (75 %) overnight. After washed with PBS (supplemented with 500 units ml^−1^ penicillin and 500 units ml^−1^ streptomycin) three times, the matrices were incubated with avidin (1 mg ml^−1^ in PBS; Sigma-Aldrich) at room temperature for 2 h. In order to compare avidin-biotin-mediated adhesion to integrin-mediated adhesion, substrata were coated with fibronectin (25 μg ml^−1^ in PBS; Sigma-Aldrich) for 2 h. Then the scaffolds were washed with PBS thrice and prepared for use.

### Cell isolation and culture

Animal procedures related to adipose tissue isolation were approved by the Shanghai JiaoTong University Ethical Committee. After the 10 % chloral hydrate (350 mg/kg) anesthesia of rats, abdominal adipose tissue (approximately 5 g) was obtained from bilateral inguinal region of SD rats and washed with PBS for 15 min. Tissue was minced by sharp dissection into 1 mm^3^ pieces, and directly exposed to PBS containing 0.1 % collagenase type I (Sigma-Aldrich, St. Louis, MO) for enzymatic digestion. After 60 min incubation at 37 °C with mild agitation (40 rpm), an equal volume of Dulbecco’s modified Eagle’s medium (DMEM, Gibco) containing 10 % FBS was added to stop enzymatic digestion. Then the mixed solution was filtered through a 70 μm nylon mesh and the filter liquor was transferred into a 15 ml centrifuge tube, finally the cellular pellet was isolated via centrifugation 1500 rpm for 10 min at room temperature. Cells were dispensed into tissue culture flasks (Corning Glass Works, Corning, NY) containing 5 ml complete medium. ADSCs were incubated in a 5 % CO_2_ incubator at 37 °C, and medium was changed every three days.

### Biotinylation of ADSCs

Biotin was applied to the biotinylation process of ADSCs according to the operating manual. Briefly, ADSCs suspension (1 × 10^5^ cells ml^−1^) was treated with biotin (Sulfo-NHS-Biotin, Pierce, Rockford, IL) at the ratio of 1 mg per 10^6^ cells. The mixture was incubated for 30 min and then washed with PBS twice by repeating centrifugation and resuspension.

### Multilineage differentiation assessment of biotinylated ADSCs

Biotinylated ADSCs were cultured in adipogenic medium comprising growth medium (low-glucose DMEM, 10 % FBS, 100 units ml^−1^ penicillin, 100 units ml^−1^ streptomycin) supplemented with 10^−6^ M dexamethasone, 0.5 mM 3-Isobutyl-1-methylxanthine, 100 μM indomethacin and 10 μg ml^−1^ insulin for an additional 21 days. Adipogenic differentiation was assessed using Oil Red O stain for adipose oil. For induction to osteocytes, biotinylated ADSCs were cultured in osteogenic medium consisting of growth medium supplemented with 10^−7^ M dexamethasone, 10 mM β-glycerol phosphate, 0.2 mM L-ascorbic acid and L-glutamine for an additional 21 days. Osteogenic differentiation was assessed using von Kossa stain for ECM calcification. For differentiation into keratinocytes biotinylated ADSCs were cultured in epidermal induction medium consisting of growth medium supplemented with 0.4 μg ml^−1^ hydrocortisone, 5 μg ml^−1^ insulin, 1 nM T3, 10 ng ml^−1^ EGF, 1 μM VD_3_, 50 μg ml^−1^ L-ascorbic acid for an additional two weeks. Epidermal differentiation was evaluated using immunostaining for cytokeratin 10 (an early marker of epidermal differentiation). Normal ADSCs incubated in the same lineage-specific medium served as positive control.

### Cell attachment assay

Four groups were designed, including Group A: ADSCs + PLCL (PLCL); Group B: ADSCs + PLCL/Pluronic (PLCL/Pluronic); Group C: biotinylated ADSCs + avidinized PLCL (AB-PLCL); Group D: biotinylated ADSCs + avidinized PLCL/Pluronic (AB-PLCL/Pluronic). All groups were incubated at 37 °C in a humidified atmosphere of 5 % CO_2_. After incubation for 10 min, 20 min, 30 min, 60 min and 5 h the solutions containing the unattached cells were discarded and the test samples were washed with PBS gently to remove the nonattached cells. Then the nanofibers were fixed with 4 % paraformaldehyde at room temperature for 15 min. After three washes with PBS, cell nuclei were stained by the 4′, 6-diamidino-2-phenylindole staining solution (DAPI, Beyotime Institute of Biotechnology, Shanghai, China) at room temperature for 15 min. All groups were observed using a fluorescence microscope. The number of cells attached on the surface of nanofibers was counted in three randomly-selected fields (40×). The data was expressed as mean number of cells per field (cells/field) ± standard deviation (SD), and was subsequently analyzed for statistically significant differences between groups. In addition, the cells were stained by Viability/Cytotoxicity Assay Kit. The mean cell spreading areas were estimated using image analysis software (Image-J, National Institutes of Health, USA) and calculated by selecting 100 cells randomly observed on the fluorescence images.

### Cell proliferation assay

After Cell attachment assay, cell growth and viability was conducted to assess the function of cells adhered on nanofibers. The ADSCs-nanofibers were incubated at 37 °C in a humidified atmosphere of 5 % CO_2_ and the culture medium was replaced every three days. After 1, 3, 5, 7, 9 and 11 days culture, the culture medium was removed and 400 μl medium containing 40 μl CCK-8 reaction solution was added to each well and ADSCs-nanofibers were incubated for 4 h at 37 °C and 5 % CO_2_. Then the medium with CCK-8 was transferred to 96-well tissue culture plate and the absorbance was read at 450 nm using a multidetection microplate reader (MK3, Thermo, USA). All experiments were carried out in triplicate. Then the growth curve was generated and the proliferation rate of ADSCs on the nanofibrous matrices was evaluated.

### Statistical analysis

Tests were done in three replicates, unless otherwise stated. All quantitative data were recorded and statistically analyzed by SPSS 19.0. Values were expressed as the mean of three replicates and standard deviation (SD). Experimental results were also analyzed by one-way ANOVA. For all statistical tests, the level of significance was set at *p* < 0.05.

## Abbreviations

PLCL: Poly(ε-caprolactone-co-lactide)
SEM: Scanning electron microscope
ABBS: Avidin-biotin binding system
FBS: Fetal bovine serum
ADSCs: Adipose-derived stem cells
PBS: Phosphate buffered saline
